# Primary vaginal endodermal sinus tumor in infants and children: experience from a tertiary center

**DOI:** 10.1186/s12887-022-03634-2

**Published:** 2022-10-07

**Authors:** Min Yin, Jiaxin Yang, Tao Wang, Sijian Li, Xinyue Zhang

**Affiliations:** Department of Obstetrics and Gynecology, National Clinical Research Center for Obstetric and Gynecologic Diseases, Peking Union Medical College Hospital, Chinese Academy of Medical Sciences and Peking Union Medical College, Beijing, China

**Keywords:** Vaginal endodermal sinus tumor, Chemotherapy, Conservative treatment, Children

## Abstract

**Background:**

The objective of the study was to analyze the clinical features, treatment, and outcomes of primary vaginal endodermal sinus tumor (EST) in infants and children treated in a tertiary center.

**Methods:**

Clinical data of patients with pathologically confirmed primary vaginal EST in our hospital from January 1997 to December 2017 were retrospectively reviewed and analyzed.

**Results:**

A total of 21 patients were included in this study. The median age at diagnosis was 11 months (range, 4–44 months). The most common manifestations were abnormal vaginal bleeding, and a polypoid mass protruding from the vagina. Chemotherapy based on PEB (cisplatin, etoposide, bleomycin) regimen was given, and serum alpha-fetoprotein (AFP) levels dropped to normal levels after 2 to 4 cycles of chemotherapy (median, 2 cycles). After 3 to 13 cycles of chemotherapy, with a median of 5 cycles, 20 patients achieved complete remission (95.2%). The median follow-up time was 80 months (range, 4-281months). At the time of the last follow-up, 19 cases were alive without disease, and the survival rate was 90.5%.

**Conclusion:**

Vaginal EST is a very rare malignant germ cell tumor and is sensitive to chemotherapy. Conservative surgery combined with PEB chemotherapy is an effective way of treatment. Serum AFP and imaging examinations can monitor the treatment response and recurrence.

## Background

Malignant germ cell tumors (GCTs) are rare tumors, accounting for 3% of all childhood malignant tumors [1]. Endodermal sinus tumor (EST) is one of the malignant germ cell tumors that usually occurs in the ovaries and testes of young patients, and the occurrence of primary EST in the vagina is extremely rare [2]. So far, more than 100 cases of vaginal YSTs have been reported [3]. According to previously reported cases, vaginal EST typically occurs in children less than 3 years of age, with a presentation of painless vaginal bleeding, often accompanied by a polypoid mass protruding from the vagina [4]. Owing to the rarity of the malignancy, universal guidelines for the diagnosis and treatment of this malignancy have not been developed. In the past, radical surgery followed by adjuvant chemotherapy and/or radiation was considered the typical treatment, sacrificing patients’ fertility [5]. Recently, conservative treatment of vaginal EST has been reported to be effective in several cases [6]. However, in the case reports of vaginal EST, data on the clinical management of this rare gynecological malignancy are limited. Herein, we retrospectively reviewed a relatively large data set comprising 21 cases of primary vaginal EST in infants and children at our institution to provide a potential framework for the diagnosis and treatment of this rare disease in the pediatric population.

## Methods

### Patient selection

Clinical data of infants and children with primary vaginal EST who were diagnosed and treated at Peking Union Medical College Hospital including patients who were referred, between January 1997 and December 2017 were retrospectively reviewed. Clinical data on patient age at diagnosis, presenting complaints, initial serum alpha-fetoprotein (AFP), imaging results, treatment regimen, stage, disease recurrence, and results of follow-up were collected from the medical records, outpatient files, or by telephone interviews.

### Treatment protocol

**Fig. 1:**
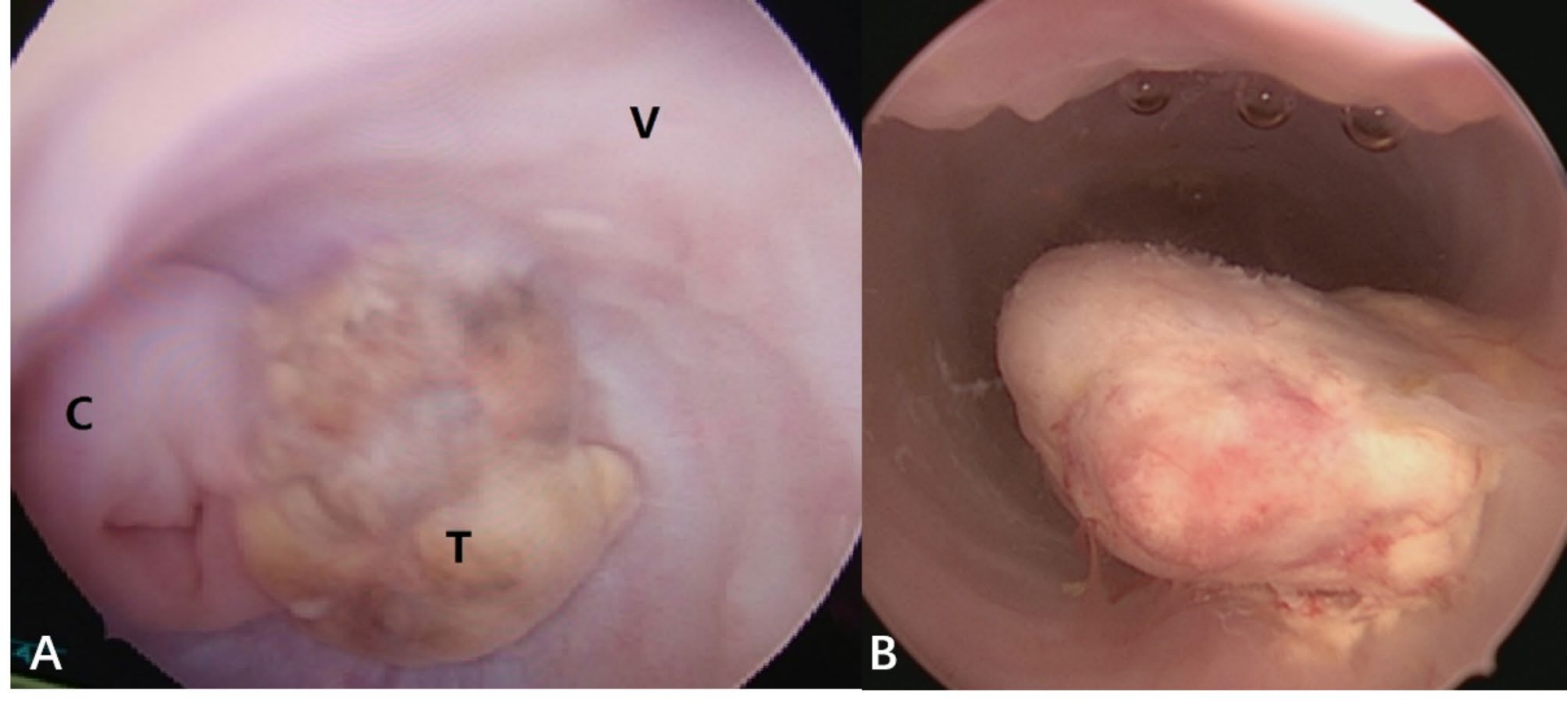
Gross aspects of vaginal endodermal sinus tumor. (A) Vaginal examination and biopsy under hysteroscope. C: Cervix; T: Tumor; V: Vaginal wall. (B) Tumor originated from the vaginal wall

Imaging examinations, including ultrasonography, computed tomography (CT), magnetic resonance imaging (MRI), and detection of serum AFP were performed to evaluate the tumor status. The diagnosis of vaginal EST was based on the pathological results of the vaginal mass or vaginal exfoliated tissues. Vaginoscopy under anesthesia was performed to assess the lesion and a biopsy was obtained if necessary to confirm the diagnosis (Fig. 1 A and 1B). Two experienced pathologists made the diagnosis independently based on the presence of the classic Schiller-Duval bodies and positive AFP staining by immunohistochemistry. Patients were staged according to the International Federation of Obstetrics and Gynecology (FIGO) criteria for vagina.

Before the initiation of chemotherapy, a subclavian venous access port was placed to facilitate the infusion of chemotherapeutic drugs. PEB chemotherapy regimen comprising cisplatin (DDP), etoposide, and bleomycin (BLM) was used as the first choice of treatment.

Specifically, cisplatin 30–35 mg/m^2^ IV daily on days 1–3 plus etoposide 100 mg/m^2^ IV daily on days 1–3 plus BLM 15 mg/m^2^ IM on day 2, day 9, day 16, repeated every 21 days. Due to the toxicity of bleomycin, the use of bleomycin was changed to 15 mg/m^2^ on days 1–2, repeated every 21 days. In addition, adverse reactions to chemotherapy were graded on a 0–4 scale for acute and sub-acute toxicity following the World Health Organization (WHO) guidelines for anti-cancer drugs. Routine blood examinations (twice a week) and hepatic and renal function tests (once a week) were performed during the treatment. Since the child cannot cooperate with the completion of the pulmonary function test, the evaluation of pulmonary function mainly relies on clinical symptoms, physical examination, and chest X-ray examination to evaluate the interstitial lung disease caused by bleomycin. If patients reached the total dose of BLM (250 mg/m^2^), which was regulated by the State Food and Drug Administration of China, or interstitial lung disease was suspected, PEV (DDP, etoposide, and vincristine), VAC (vincristine, dactinomycin, and cyclophosphamide), or PE (DDP and etoposide) chemotherapy regimens were used alternatively.

Serum AFP was closely monitored after each course of chemotherapy or before the start of the next course of chemotherapy to evaluate the tumor response. Chemotherapy was considered effective if the serum AFP level decreased logarithmically with every cycle. It was considered a serologic complete remission (sCR) when serum AFP level reduces to the normal range (< 20 ng/mL, the normal reference level in our hospital). After achieving sCR, consolidation chemotherapy was continued for two or more cycles accordingly. Comprehensive evaluations including imaging, vaginal examination, and necessary biopsies were performed. Complete remission (CR) was considered if the AFP was normal and the pathology was negative.

### Follow-up

All patients underwent regular follow-up surveillance after achieving CR. Abdomen and rectal examinations, serum AFP, and imaging examinations were performed monthly during the first year, every 2 months during the second year, every 3 months during the third and fourth years, and every 6 months during the fifth year and thereafter. The intravenous port could be removed about 1 year after chemotherapy. Recurrence was defined as either an abnormal serum AFP level or a finding of the lesion. Patients were followed through December 31, 2020.

### Statistical analysis

The time between the date of diagnosis and disease recurrence or the date of the last follow-up was defined as disease-free survival (DFS). Overall survival (OS) was defined as the time between the date of diagnosis and death or the date of the last follow-up. The disease-free and overall survivals were calculated using the Kaplan-Meier method.

## Result

### Clinical characteristics

A total of 21 patients were enrolled in this study, including 17 patients who were initially treated in our hospital and 4 patients who were transferred to our hospital after surgery and/or chemotherapy in other hospitals. The clinicopathological characteristics, treatment regimen, and outcomes of the patients were listed in Table 1. The median age at diagnosis was 11 months (range, 4–44 months). Among the 21 patients, 14 patients (67%) were younger than 1 year old, and only 1 patient was older than 3 years old. Twenty patients were delivered at term and only 1 patient (Case 14) was born prematurely as one of the twins at 32 weeks, with a birth weight of 1850 g. None of the mothers of the patients had a history of oral contraception and diethylstilbestrol or radiation exposure. The most common presenting symptom was abnormal vaginal bleeding, and other complications at presentation included a polypoid mass protruding from the vagina (n = 11), anemia (n = 3), and fever (n = 2). The median tumor size was 4 cm (range, 1–9 cm). Serum AFP levels were significantly elevated in all patients, and the median level was 4638 ng/mL (range, 251-54000 ng/mL). Twenty patients (95.2%) were FIGO stage I, and only 1 patient (Case 19) was stage II, with intrapelvic metastases (internal iliac vein tumor thrombus).

**Table 1:** Clinical features and treatment of 21 cases of primary vaginal endodermal sinus tumor

Case number	Age at diagnosis (month)	Initial serum AFP (ng/ml)	Tumor size (cm)	FIGO stage	Treatment regimen	Cycles before sCR	Cycles after sCR	Relapse location	Follow-up outcomes	Follow-up time (month)
1	20	15,100	2	I	PEB*5 + PEV*3 + VAC*1	3	6	-	NED	281
2	44	646	5	I	PEB*5 + PEV*1 + VAC*1	2	5	-	NED	278
3	11	13,890	5	I	PEB*5	3	2	-	NED	206
4	11	10,268	5	I	PEB*6	3	3	-	NED	122
5	13	768	2	I	PEB*4	2	2	-	NED	118
6	11	1110	6	I	PEB*5	2	3	-	NED	111
7	8	4638	4	I	PEB*4	2	2	-	NED	100
8	10	8754	4	I	PEB*1 + PEV*3 + VAC*1	2	4	-	NED	96
9	7	29,887	6	I	PEB*5	3	2	-	NED	94
10	9	365	1	I	PEB*4	2	2	-	NED	80
11	11	1289	4	I	PEB*4	2	2	-	NED	71
12	28	251	1	I	PEB*4	2	2	-	NED	72
13	9	19,766	6	I	PEB*5 + PE*1	4	2	-	NED	69
14	8	2626	2	I	PEB*4	3	1	-	Dead of infection and heart failure	4
15	11	3293	3	I	PEB*5	3	2	Vagina (7 months after initial treatment)	NED	56
16	10	32,015	4	I	PEB*5	3	2	Vagina (3 months after last chemotherapy)	NED	49
17	4	1526	3	I	PEB*4	2	2	-	NED	44
18	36	54,000	7	I	Transabdominal vaginal lesion resection, PEB*10	3	7	-	NED	122
19	15	1000	5	II	Transabdominal vaginal lesion resection + internal iliac venous embolectomy, NVEB*1 + PEB*2	1	2	-	NED	62
20	32	33,147	9	I	CEB*6 + PEV*2 + PEB*5	-	-	-	DOD	10
21	10	6977	4	I	Transabdominal vaginal lesion resection, VPEB*2 + CEB*1 + PEB*2	3	2	-	NED	51

**Abbreviations**:

sCR, serologic complete remission; PEB, cisplatin, etoposide and bleomycin; PEV, cisplatin, etoposide and vincristine; VAC, vincristine, dactinomycin and cyclophosphamide; PE, cisplatin and etoposide; NVEB, nedaplatin, vindesine, etoposide and bleomycin; CEB, carboplatin, etoposide and bleomycin; VPEB, vincristine, cisplatin, etoposide and bleomycin; DOD, die of disease; NED, no evidence of disease

### Treatment efficacy

As is shown in Table 1, the first 17 patients who were initially diagnosed with vaginal EST in our hospital, were treated with chemotherapy alone. Among them, 13 patients only underwent PEB chemotherapy, while 4 patients underwent initial PEB chemotherapy and subsequent PEV/VAC/PE chemotherapy after the onset of suspected interstitial lung disease or reaching the total dose of BLM.

Case 18 to Case 21 were transferred to our hospital after surgery and/or chemotherapy in other hospitals. Case 18 underwent vaginal lesion resection and 10 cycles of PEB chemotherapy at the local hospital. Twenty-three months after chemotherapy, she was transferred to our hospital due to a suspected recurrence on both ovaries. Then she underwent laparoscopic exploration, identifying cystadenomas on both ovaries instead of a recurrence of EST. Case 19 underwent transabdominal vaginal lesion resection, pelvic lymphadenectomy, and internal iliac venous embolectomy in another hospital. The pathologic examination revealed tumor involvement in the internal iliac vein. She received 1 cycle of NVEB chemotherapy (nedaplatin, vindesine, etoposide, and BLM). Then she was referred to our hospital and subsequent PEB chemotherapy was administered. Case 20 was diagnosed with vaginal EST by biopsy and underwent 6 cycles of CEB (carboplatin, etoposide, and BLM) and 2 cycles of PEV, but the tumor was uncontrolled. She was transferred to our hospital for 5 cycles of PEB. Unfortunately, the tumor developed progressively and eventually, she died of disease. Case 21 was transferred to our hospital and received PEB chemotherapy after transabdominal vaginal lesion resection and VPEB (vincristine, DDP, etoposide, and BLM) and CEB chemotherapy in another hospital.

Serum AFP levels dropped significantly after chemotherapy except in Case 21, and patients achieved sCR after 2 to 4 cycles of chemotherapy (median, 2 cycles). After sCR, several cycles of chemotherapy were added to consolidate the treatment effect and reduce the risk of recurrence. Initially, due to inexperience with the conservative treatment of vaginal EST, 5 to 6 more cycles of chemotherapy were given. Subsequently, we reduced the consolidation therapy cycles to 2 cycles in 13 patients. At the end of completing the treatment, the median cycle of chemotherapy was 5 cycles (range, 3–13 cycles). Finally, 19 patients were reassessed by ultrasound, MRI, or CT. Thirteen patients with suspect residual tumors underwent a second biopsy, and the pathology of all the samples showed negative for malignancy. To sum up, the overall chemotherapy effective rate was 95.2%.

### Chemotherapeutic adverse reactions

Twenty-one patients received a total of 102 courses of chemotherapy in our hospital. Chemotherapeutic adverse reactions included anorexia, fatigue, bone marrow suppression, liver function damage, and interstitial lung disease. Myelosuppression was the most common adverse reaction during treatment. There were 15 patients (71.4%) with grade 3 to 4 neutropenia and granulocyte colony-stimulating factor was used. One patient developed suspicious pulmonary interstitial lesions after one cycle of PEB chemotherapy, so PEV and VAC were administered thereafter.

### Oncological and long-term follow-up outcomes

**Fig. 2:**
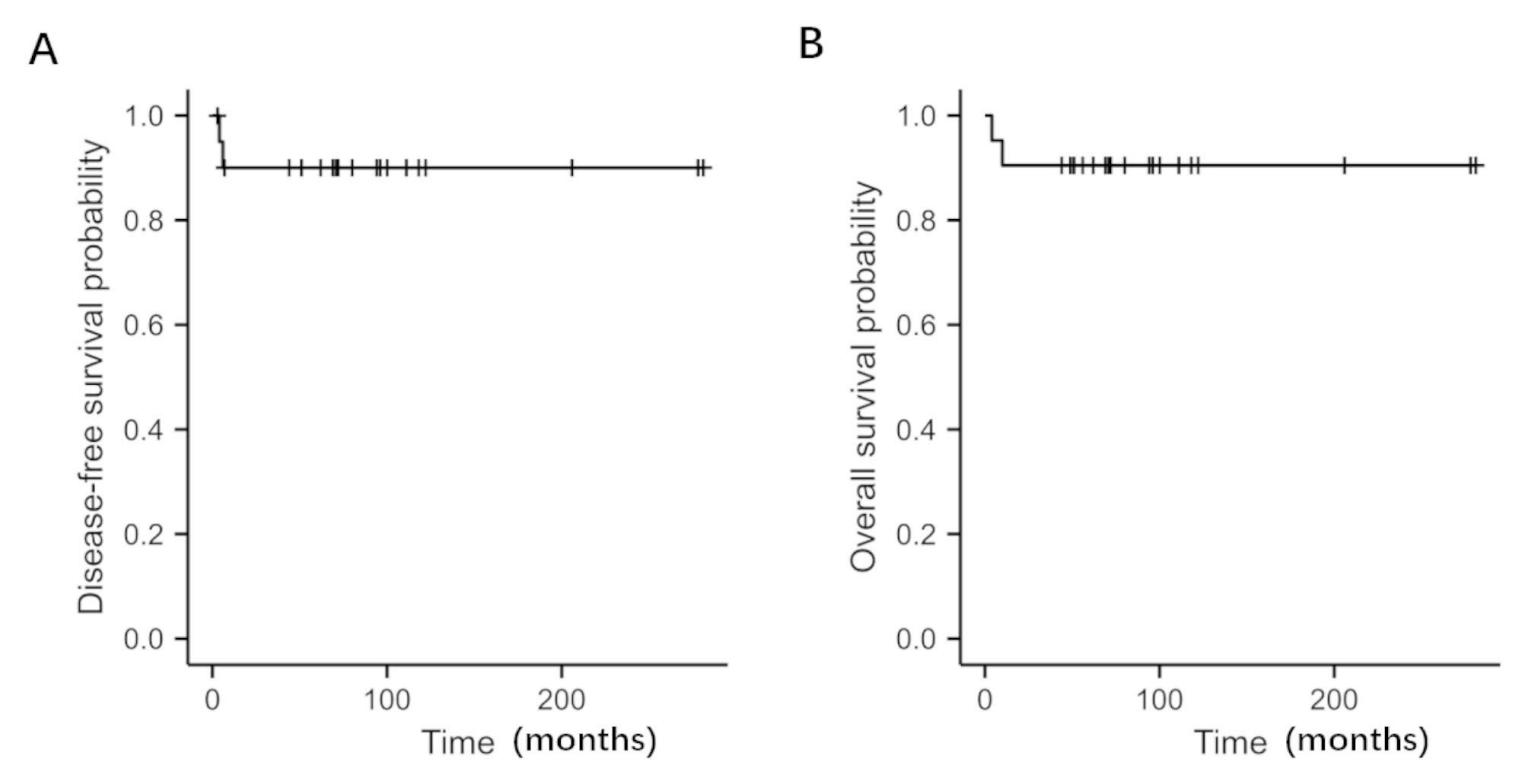
Kaplan-Meier curve of 21 patients with vaginal EST. (A) Disease-free survival. (B) Overall survival

As of December 31, 2020, median follow-up time was 80 months (range, 4-281months). At the time of last follow-up, 19 patients (90.5%) were alive without evidence of disease. The detailed follow-up information and survival data were summarized in Table 1. The Kaplan-Meier curves of DFS and OS were displayed in Fig. 2 A and Fig. 2B. Since some patients were followed for less than 5 years, we calculated 2-year DFS and 2-year OS, which was 80.95% and 90.48%, respectively.

Two cases experienced recurrence localized in the vagina. Case 15 developed a vaginal recurrence 7 months after CR and 5 cycles of PEB were administered additionally. Then she achieved CR again, living without evidence of disease. Case 16 developed a vaginal recurrence 3 months after the termination of chemotherapy. She underwent laparoscopic pelvic tumor resection followed by PEB and PEV chemotherapy, achieving CR again. One case died during the treatment. Case 14 was born prematurely as one of twins at 32 weeks. She was diagnosed with vaginal EST at 8-months old after a vaginal biopsy because of vaginal bleeding. Her serum AFP returned to normal after 3 cycles of PEB chemotherapy. After the 4th PEB cycle, she developed aspiration pneumonia after vomiting, followed by acute dyspnea and heart failure, and eventually died of respiratory and circulatory failure. During the long-term follow-up time, the level of serum AFP was kept in the normal range. Five patients reported menarche, 2 of whom underwent menarche at the age of 13 years and 3 patients at 11 years old.

## Discussion

Malignant germ cell tumors account for only 3% of malignancies in the pediatric population, and primary vaginal ESTs only constitute 3–8% of all GCTs [7]. The most common vaginal malignant tumor is embryonal rhabdomyosarcoma (RMS), followed by vaginal EST and clear cell carcinoma. However, in China, ESTs are the most common type of vaginal tumor, followed by RMS [8]. EST of the vagina occurs primarily in infants younger than three years of age. Early ESTs may be asymptomatic, but with the development of the disease, painless abnormal vaginal bleeding or abnormal vaginal discharge may occur [9].

Since vaginal examination is difficult in young children, various imaging examinations can be used to assess the location and extent of lesions. CT and MRI are considered non-invasive diagnostic tools for vaginal RMS and EST [10]. Serum AFP levels are essential for the diagnosis of malignant germ cell tumors, especially ESTs [11]. Moreover, serum AFP is considered a reliable marker to evaluate treatment response and remission status [12]. However, the gold standard diagnosis requires pathological examination. Vaginoscopy under anesthesia can fully assess the lesion and obtain a biopsy for pathological diagnosis. Schiller-Duval bodies are characteristic of the ESTs, showing a loose reticular mesh network and papillae-like structures consisting of a central vascular core lined by a single layer of cells [9]. And most ESTs are immunohistochemically positive for AFP.

**Fig. 3:**
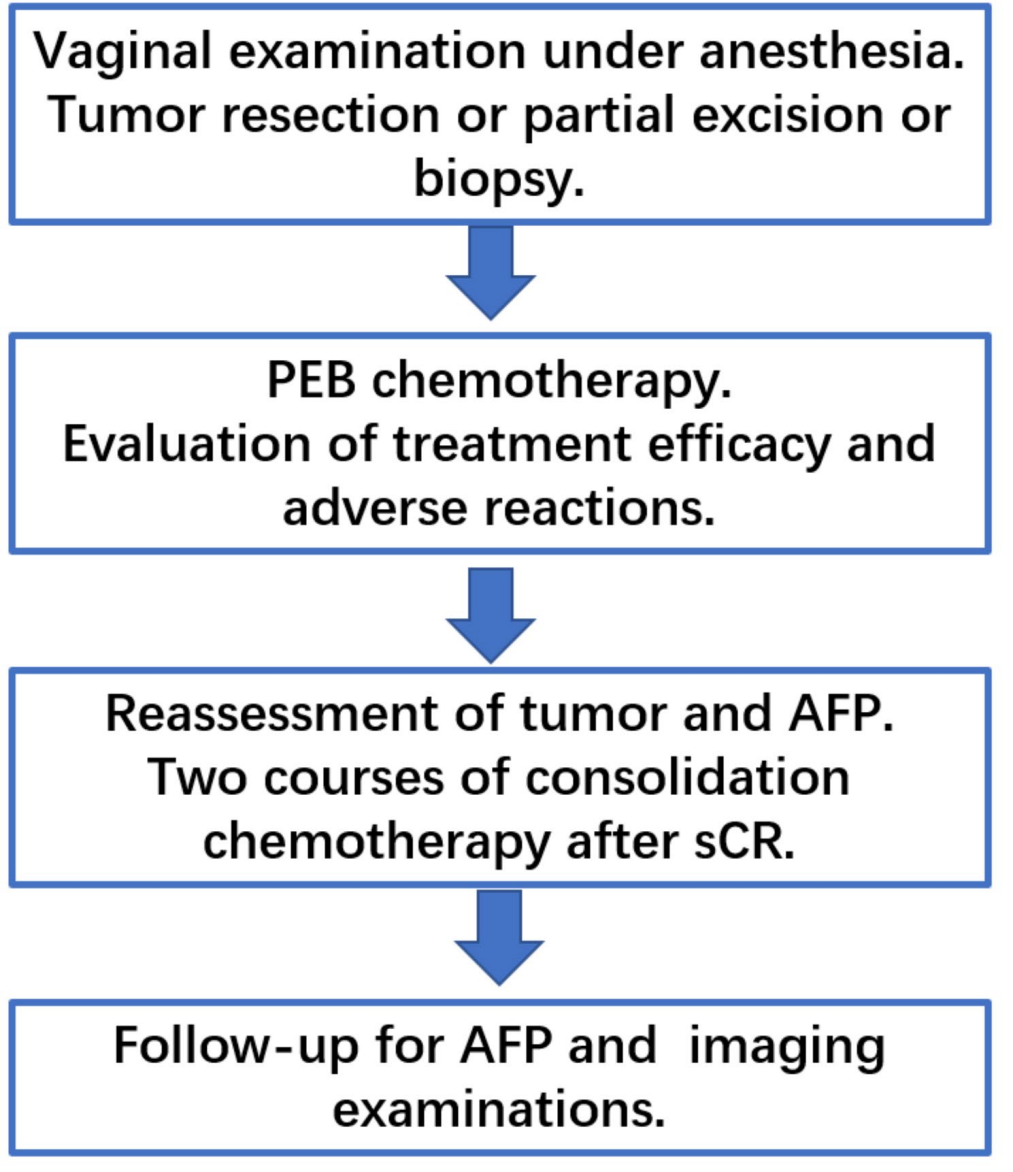
Flow chart of treatment of primary vaginal EST in children

Vaginal EST is a highly malignant tumor that not only aggresses directly but also metastasizes by hematogenous and lymphatic pathways [13]. As early as 1965, radical surgery and/or irradiation were the main treatments, sacrificing sexual function and fertility. Additionally, other sequelae such as aseptic femoral head necrosis and abnormal pelvic bone growth were also documented [14]. In a literature review comprising 137 cases of vaginal ESTs, treatment before the year 2000 involved more radical surgical approaches but 40% of the reported cases resulted in death [3]. Since the 1970s, cisplatin-based multiagent chemotherapy has significantly improved outcomes in children with malignant GCTs [15]. Treatment of vaginal EST has transitioned from aggressive surgery to conservative treatment with chemotherapy after the confirmation of a pathological diagnosis. In 1999, our center reported the first case of conservative treatment of vaginal EST in China. Later, we published the treatment experiences of six cases, and all patients were managed successfully by PEB combination chemotherapy alone without radical surgery [16]. With the accumulation of treatment experience, our center summarized the treatment process of vaginal EST and continuously improved it (Fig. 3). It is reported that since 2000, half of the patients received only chemotherapy, and more than 90% of the children were still alive and showed no evidence of disease up to 25 years after treatment with chemotherapy alone [3].

As for the residual lesions after achieving sCR, there is no consensus. Some studies recommend partial vaginal resection for residual lesions. Meng et al. claimed that surgical resection could not only remove residual lesions but also confirm the presence of active tumor cells through pathology so as to provide a basis for subsequent treatment [17]. Tang et al. considered that chemotherapy alone was more suitable for simple yolk sac tumors, but tumors with other mixed germ cell components should be treated with chemotherapy combined with local surgery [18]. In our study, 13 patients with suspect residual tumors after sCR underwent a second biopsy, and none of the pathologies showed active tumor cells.

Significant improvements in pediatric cancer survival rates increase the number of long-term survivors. As a result, their subsequent quality of life, including fertility, is becoming increasingly important. The extent of fertility damage is determined by the chemotherapy agents and the age of the patient at the time of treatment [19]. Alkylating-like agents, such as cisplatin and carboplatin, are particularly gonadotoxic. Patients treated with vincristine, bleomycin, and vinblastine have a lower risk for infertility [20]. Reinmuth S et al. investigated the relationship between chemotherapy and infertility in survivors of pediatric cancer. Etoposide, particularly ≥ 5000 mg/m^2^ in women, and carboplatin and/or cisplatin in both sexes appeared to have an independent risk for infertility [21]. But chemotherapy before puberty showed less possibility of infertility compared to treatment during or post-puberty [22]. In addition, Wallace et al. estimated the risk of developing subfertility depending on the illness and the established treatment regimens. Germ-cell tumors with gonadal preservation and no radiotherapy were grouped in low risk of developing subfertility [23]. In our study, the follow-up time of children is not long enough, so we are unable to assess the influence of these treatments on patients’ puberty development, sexual ability, and future fertility, which was a limitation of our study.

## Conclusion

In conclusion, our study shows that vaginal ESTs occur almost exclusively in girls under the age of 3 years, with vaginal bleeding as the first symptom of the disease. Conservative surgery combined with PEB chemotherapy should be considered as an effective treatment for patients in the early stage. Serum AFP is a reliable marker for diagnosis, treatment response assessment, and recurrence monitoring. Long-term follow-up is warranted to evaluate the impact of chemotherapy in childhood on fertility in adulthood.

## Data Availability

All data generated or analyzed during this study are included in this published article.

## Abbreviations

GCTs: germ cell tumors.
EST: endodermal sinus tumor.
AFP: alpha-fetoprotein.
CT: computed tomography.
MRI: magnetic resonance imaging.
DDP: cisplatin.
BLM: bleomycin.
ADC: apparent diffusion coefficient.
